# Contribution of a Real Depth Distance Stereoacuity Test to Clinical Management

**DOI:** 10.1155/2009/343827

**Published:** 2009-07-05

**Authors:** B. J. Young, H. Sueke, J. M. Wylie, S. B. Kaye

**Affiliations:** Department of Ophthalmology, St Paul's Eye Unit, 8Z Link, Royal Liverpool University Hospital, Prescot Street, Liverpool L7 8XP, UK

## Abstract

Measurement of Stereopsis forms an important part of the clinical assessment of patients with disorders of ocular motility. The introduction of a real depth distance stereoacuity test (FD2) was evaluated in clinical practice and to what extent the introduction affected clinical management. Seventy-three patients under evaluation before and following the introduction of the test were included. Combined thresholds were measured at near using the Frisby and TNO test and at distance using the FD2. Fifty healthy controls were included. Forty-five patients demonstrated Stereopsis using the FD2 and 23 of these had a change in their management based in part on their responses using the FD2. Patients with evidence of Stereopsis using the FD2 were significantly more likely to have change in their management than expected from the whole sample (*P* = .02). The introduction of a real depth distance stereoacuity test into clinical practice contributed to a change in management when used in conjunction with other tests. The usefulness of the FD2 is limited by its range at 6 m. Use at closer distances necessitates the calculation of binocular threshold from the combined and monocular threshold.

## 1. Introduction

Stereopsis is routinely measured at near as part of the clinical assessment of patients with disorders of ocular motility and strabismus. Recently the FD2 [1, 2] has been introduced in order to measure stereopsis at distance. The present of stereopsis at a given distance indicates that a patient has binocular control at that position. Binocular control can vary with distance depending on the state of the patient's ocular alignment. It is important therefore, to assess stereopsis at different distances in the clinical environment.

Stereopsis is usually reserved for the impression of depth arising from binocular disparity [3–5]. There are, however, many other binocular (vergence, luminance, chromostereopsis, texture, etc.) and monocular (parallax, size, luminance, etc.) cues that provide important depth information [6, 7]. These cues vary in the different stereoacuity tests, whether real object (Frisby, VDS, FD2) or simulated (TNO, BVAT, Titmus). The sum of these cues contributes to a combined threshold of depth discrimination (CT) [8] which is actually what is measured by most available stereoacuity tests. It is because these cues vary in different tests and with different viewing distances and gaze positions, that differences in measured “stereoacuity” thresholds are often found.

Until the introduction of distance stereoacuity tests, assessment of binocular alignment when viewing distant objects relied upon the absence of a detectable heterotropia and the response to Bagolini striated lenses or the Worth 4 Dot test. Neither test, however, provides sufficient information to demonstrate the present of stereopsis. The FD2 is a real object or depth rather than simulated stereoacuity test. Although it is, therefore, not prone to disassociative stimuli, because it is a real object test, it is prone to monocular cues and limited in its range.

The use of the FD2 to evaluate the success of exotropia surgery has been evaluated [9] but it is unclear, however, whether the use of a real depth test such as the FD2 contributes to the actual clinical management of patients with disorders of ocular motility. The contribution of the FD2 to the management of a group of patients was therefore investigated.

## 2. Methods

A retrospective case note review of 73 consecutive adult patients with disorders of ocular motility who, had been under review before and following the introduction of the FD2 were included. They were divided into 4 groups according to whether their depth discrimination could be measured for; both near and distance, only at one distance, or not at either distance. Patients in each group were then assessed according to whether there had been a change in their management and whether this was based in part on the information provided by the FD2. A prospective study was undertaken on 50 healthy individuals to determine the monocular thresholds and normative data of the stereoacuity tests that had been used.

### 2.1. Ocular Alignment and Visual Acuity

Ocular deviations were measured (using alternate prism and cover test) in prism dioptres (rounded to nearest 2PD for deviations up to and including 20PD, and the nearest 5PD for deviations greater than 20PD). Monocular near and distance best corrected visual acuity (BCVA) was measured at 6 m using Snellen chart and at 0.33 m, using a near reading type.

### 2.2. Measurement of Depth Discrimination

Stereoacuity tests were used according to the manufacturer's instructions. Depth discrimination thresholds were measured at near (between 0.3 m and 0.8 m) using either the TNO or Frisby stereoacuity tests in the normal reading position (i.e., on downgaze) and at distance (6 m) using the FD2. The range of thresholds for the Frisby for each of the 3 plates can only be varied by changing the viewing distance, and the measured thresholds were at the distances specified between 0.3 m and 0.8 m by the manufacturer. The FD2 comprises of a box containing four back illuminated shapes mounted on rods, in a transparent support frame. Each of the shapes can be moved so that one of them is set nearer than the other three shapes. The rods are marked, so that disparity can be set between 5 and 50 seconds of arc in steps of 5 seconds. The FD2 was positioned at the correct measured height for each subject's eye level, and patients were asked not to move their head during testing. The subjects were required to identify three out of four correct choices at a given disparity until the patient reached threshold. In the control group thresholds of depth discrimination were measured with both eyes open (combined threshold, (CT)) and then with one eye occluded (monocular threshold, (MT)) for both the near Frisby and FD2 at 6 m, 3 m, and 1 m. The binocular threshold (BT) was calculated, according to the function, BT  = (1/CT-1/MT)^−1^ [6]

### 2.3. Analysis

Chi-squared test was used to test for a difference between the patient groups. The logarithm of the depth discrimination threshold (combined, monocular, and binocular) was used for the analysis. For comparison between the stereoacuity tests in the control group a repeated measures model was used.

## 3. Results

### 3.1. Control Group

50 healthy subjects (mean age 38 years (12)) were included. Using the FD2, MT was not measurable at 6 m. At 3 m MT was measurable in 10% and at 1 m in 60% of subjects (Table 1). There were linear but relatively weak associations between the FD2 at 6 m and the Frisby (*R* ^2^ = 0.40, *P* < .001) and TNO (*R* ^2^ = 0.28, *P* < .001) tests. There was also a significant association between age and depth discrimination (both near *P* = .003 and distance *P* = .002).

### 3.2. Patients

73 adult patients were included (mean age 58.3 years (19.5)). There were 39 patients whose depth discrimination thresholds could be measured at both distances (FD2 and near test positive), and 6 patients whose depth discrimination could not be measured with either test (FD2 and near test negative). There were 22 patients whose depth discrimination thresholds could be measured at near only, and 6 patients whose depth discrimination could be measured at distance only. Nine of the patients who had incongruous results with the near and FD2 tests had a change in management. Overall 23 patients had a change in management, the FD2 contributing in part to this decision (Table 2).

### 3.3. FD2 Positive, Near Negative

Six patients (68–73 Table 2) had stereoacuity measurable only on the FD2. Four of these patients had torsional misalignment more evident for near on depression. Two patients had a variable angle at near with no measurable near depth discrimination. Prior to the introduction of the FD2 these six patients had been thought to have no useful stereopsis based on near stereoacuity tests placed in the normal reading position, that is, downgaze. Using the FD2 however they had measurable depth discrimination (mean 38.3 seconds) at 6 m with a prism in place. Prisms were therefore prescribed.

### 3.4. FD2 Negative, Near Positive

Patients 20 and 22 (Table 2) had no stereopsis measurable at distance. The first patient, had his prism reduced on his distance spectacle segment, as using the FD2 no achievable stereopsis could be demonstrated at distance. A second patient, who had previously demonstrated 15 seconds with the FD2, lost this function due to a change in his ocular alignment associated with deterioration of his thyroid eye disease. This patient did not show any change in his near depth threshold, which remained at 55 seconds. A third patient (number 21) had a decompensating exophoria and was found to have lost their distance stereopsis (previously 50 seconds) and 2 lines of BCVA (from 6/5 to 6/9) but with no change in their near stereoacuity. This patient was found to have had a recurrent retinal detachment and was therefore excluded from further analysis.

### 3.5. FD2 and Near Positive

Patients 48 to 61 (Table 2) had stereoacuity measurable on both the FD2 and near tests. In ten of these fourteen patients, the FD2 was used to determine at what distance a prism was required and how much prism was necessary to gain stereoacuity. In patients undergoing surgery for strabismus, the FD2 was used to confirm the presence of quantifiable binocular function at the corrected angle for distance in free space.

Overall, patients who had evidence of depth discrimination stereopsis using the FD2 were significantly more likely to have some modifications to their clinical management than expected from the whole patient sample (*P* = .023). This was particularly so for patients who had evidence of depth discrimination on the FD2 but not using the near tests (*P* < .001).

## 4. Discussion

The most commonly used stereo tests in clinical practice are usually viewed at near. In addition, many adult patients wear bifocals or varifocals requiring that the near stereo tests be placed in their normal reading position. Distance stereoacuity using the FD2 is measured at eye level. One therefore cannot assume that because an individual has stereoacuity at near or in a reading position, that they will also have stereoacuity at distance in the primary position. For example, in this study some patients achieved 25 seconds using the FD2 but only achieved 120 seconds using the Frisby at 0.4 m. It is unclear whether this difference arose because of changes in ocular position or differences in the inherent monocular and binocular cue content in each test. This is apparent in the relatively poor correlation between near tests and FD2.

Importantly we found that for the patients in this study, the demonstration of stereopsis at distance contributed to a change in their management. Although it is difficult to be sure of the true contribution of the FD2 to the change in management given that a change in condition could have occurred before and after introduction of FD2, this is unlikely as most of the patients' ocular motility was relatively stable. Similarly whilst it is possible that a similar proportion of patients may have had a change in their management in the absence of the results of the FD2, this is also unlikely, given that the management decisions were influenced by the results of the FD2.

The relatively poor association between the TNO, Frisby, and FD2 for the healthy control where the maximum association was 40% and for patient group, where there was no significant association, is likely to reflect the differences in the monocular and binocular cue content of these tests. For example, although vertical disparity, accommodation, and vergence are potentially important binocular cues for near tests, they may play less of a role in distance stereoacuity tests [6, 10–18]. These differences in monocular and binocular cues are also likely to account in part for the differences in measured thresholds. For example, although all of the patients who were near positive but distance negative had near stereoacuity values greater than 50 seconds, there were many patients who had near and distance stereoacuity with distance stereoacuity less than 50 seconds, despite their near stereoacuity values being greater than 50 seconds of arc.

The targets used in the near stereoacuity tests have minimum and maximum thresholds of 15 seconds and 1980 seconds (TNO) and 20 seconds and 600 seconds (Frisby). In contrast the FD2 at 6 m has a minimum and maximum threshold of 5 seconds and 50 seconds. This together with the noncontinuous or stepwise increments in all of these stereoacuity tests means that there is little overlap between the near tests and the FD2.

This limitation may account for those patients who had thresholds greater than 50 seconds with near tests but who had no demonstrable stereopsis using the FD2 at 6 m. Shortening of the viewing distance to 3 m extends the maximum threshold with the FD2, but also leads to an increase in monocular cues [19], see Table 1. Monocular cues facilitate depth perception and form an integral part of the stereo mechanism [6, 20], and it has been shown that monocular cues may contribute to depth even when they do not reach threshold [6]. Previous investigations of the FD2 have reported a problem with the monocular cue content [21] thus necessitating either a change in protocol or measurement of the monocular threshold. As discussed, although stereopsis is usually reserved for the impression of depth arising from binocular disparity [3–5, 8] horizontal and nonhorizontal [12, 20] other binocular (vergence, luminance, chromostereopsis, texture, etc.) and monocular (parallax, size, luminance, etc.) cues provide important depth information [6, 7]. These cues summate in any stereoacuity test so that what is measured is a combined threshold of depth discrimination [8]. It has, however, been shown that subtraction of the monocular threshold from the combined threshold allows calculation of the actual binocular threshold (BT) of depth discrimination [6] which, is what is of interest when assessing binocularity. Thus when measuring a patient's level of stereoacuity if monocular cues reach threshold, rather than abandoning the test, the monocular threshold should be subtracted from the binocular threshold according to the function, BT  = (1/CT-1/MT)^−1^ [6, 7, 19, 20].

Other distance stereoacuity tests have been introduced such as the Mentor Binocular Video Acuity Tester (BVAT) [22–24] and the Variable Distance Stereotest (VDS) [3, 6]. The importance of measuring stereoacuity at distance has been particularly well demonstrated using the BVAT and VDS. The BVAT is a simulated distance stereotest. The test requires the use of goggles that are potentially disassociative and do not facilitate the correction of any deviation using prisms. The BVAT being a simulated test has zero parallax, which implies that the object is flat. The VDS based on the classic three-rod arrangement of Helmholtz [25], and although providing a continuous measurement of stereoacuity in seconds of arc has never been commercially available.

The introduction into clinical practice of the FD2 distance stereotest was found to contribute to clinical decision making which otherwise may not have occurred using only near stereoacuity tests. The FD2 is however limited by its range at 6 meters and using the test at shorter distances necessitates measurement of MT with calculation of the BT as described above. Despite these limitations measurement of stereoacuity at distance is an important aspect in the evaluation of patients with disorders of ocular motility.

## Figures and Tables

**Table 1:** Depth discrimination thresholds of control subjects using the near stereoacuity tests, Frisby, TNO, and the FD2 at 1 m, 3 m, and 6 m. Combined (CT), monocular (MT), and binocular (BT) depth discrimination thresholds. Mean, standard deviation (SD) and median in logarithm of arc seconds. Range of test and subjects in arc seconds. Not measurable (NM).

Test	Mean CT (SD) Median (Log seconds)	Range of CT (seconds)	Measurable range of stereoacuity test (seconds)	Mean BT (SD) Median (Log seconds)	Mean MT (SD) Median (Log seconds)
TNO	1.75 (040) 1.78	15–1980	15–1980	1.75 (0.40) 1.78	NM
Frisby	1.74 (0.34) 1.83	20–170	20–600	1.74 (0.34) 1.83	NM
FD2 6M	1.14 (0.28) 1.18	5–50	5–50	1.14 (0.28) 1.18	NM
FD2 3M	1.45 (0.23) 1.30	20–160	140–200	1.47 (0.25) 1.30	2.34 (0.2) 2.30
FD2 1M	2.28 (0.39) 2.26	182–558	952–2042	2.32 (0.10) 2.31	3.13 (0.15) 3.14

**Table 2:** Patients who had a change in clinical management following introduction of FD2. Patients 68–73 had evidence of stereopsis using the FD2 but not with near tests, patients 20–22, stereopsis with the near tests but not FD2 and patients 48–61 evidence of stereopsis with both the FD2 and near tests, respectively. Patient 21 was excluded from further analysis as the change was due to retinal detachment. Summary of the change in management is included in the right-most column. Diagnosis is included where established. Where no specific diagnosis established, extraocular muscle deviation recorded. Prism dioptres (pd), intermittent heterotropia (T), esophoria (EP), esotropia (ET), hyper or hypotropia (HT), hyper or hypophoria (HP), base-out (BO), and base-in (BI) prism, lateral rectus (LR), superior rectus (SR), superior oblique (SO), medial rectus (MR), inferior rectus (IR), right (R) and left (L), underaction (u/a), thyroid eye disease (TED).

Patient	Symptom	Deviation		Diagnosis/lesion	FD2(sec)	Near(sec)	Change in management
		Distance	Near				
68	Diplopia on depression	3pd HP, 5^0^ cyclotorsion	4pd HT, 15^0^ cyclotorsion	(L)IVn paresis	50 with prism	nil	Prism accepted over entire varifocal
69	Intermittent diplopia	3pd HT	2pd ET, 2pd HT	Bilateral IR u/a. Pineal germinoma	25 with prism	nil	Given weakest prism possible to achieve distance stereo.
70	Diplopia	10pd ET, 5pd HT	16pd ET, 6pd HT cyclotorsion	LR and (L) SR and (R) SO u/a	25 with prism	nil	BO prism achieved dist stereo, but not for near
71	Increasing diplopia on downgaze	12pd E(T)	8pd E(T), 14pd ET on downgaze	Bilat VIn paresis. Acoustic neuroma	50 with prism	nil	Increased BO prism for distance to achieve distance stereo.
72	Diplopia	7pd HT	7pd HT, 7pd XT cyclotorsion	(L) IVn paresis	30 with prism	nil	Vertical prism accepted for distance.
73	Reversal of diplopia at near with prism	4pd HT	2pd HT cyclotorsion	(R) SR, (L) SO u/a	50 with prism	nil	Vertical prism accepted only for distance.
20	Intermittent diplopia	10pd ET, 4pd H(T)	2pd EP	Bilateral VIn (L) SR u/a	nil with prism	120 with prism	No diplopia with reduced prism. FD2 negative with deviation corrected
21	Intermittent diplopia	8pd XP	25pd XP	Decompensated XP Retinal detachment	nil	240	FD2 positive previously. Referred to vitreo retinal team.
22	Intermittent diplopia	4pd EP(T)	2pd EP	TED. Proptosis L > R	nil	55	Orbital decompression. FD2 positive previously.
48	Diplopia on depression	7pd HP	7pd H(T)	IR u/a	30	300 with prism	Prism only on reading segment
49	Intermittent diplopia	6pd E(T)	2pd EP	(L)VIn	50 with prism	150	Prism over distance segment
50	Intermittent diplopia	2pd H(T)	10pd XP	TED	30 with prism	85	Prism over distance segment
51	Asymptomatic	2–4 pd E(T)	6pd XP	(R)VIn	50 with prism	480	Binocular with prism over distance segment
52	Intermittent diplopia	8pd E(T)	4pd EP	Bilat VIn	50 with prism	60	Increased prism to achieve distance stereoacuity
53	Resolving diplopia	6pd EP	2pd EP	(L)VIn resolving	15	55	Binocular without prism
54	Intermittent diplopia for near objects	12pd XP	35pd X(T)	Decompensating XP	50 without prism	600 with 10pd BI prism	Prism removed from distance segment
55	Blurred vision.	0 with spectacles	4pd XP to variable ET	Intermittent accommodative spasm	15 with spectacles	60	Accepted spectacles to achieve binocularity.
56	Intermittent diplopia for distant objects	2–4pd HT	2pd HP	(R) SR u/a	15 with prism	60	Binocular only with prism
57	Diplopia (monocular and binocular)	2pd EP	6pd X(T)	MR u/a Polyopia/correctopia	15 with and without prism	110	Removed prism for distance
58	Diplopia at near	4^⋀^XP	10^⋀^X(T)	Decompensating XP	40	1980 without, 85 with prism	Separate reading glasses with prism incorporated
59	Intermittent diplopia	6^⋀^ET	4^⋀^EP	Bilateral VIn	50 with prism	120	Not aware of diplopia but binocular only with prism
60	Diplopia	6^⋀^H(T)	4^⋀^HP	Partial IIIn	30 with prism	150	Binocular with prisms. For strabismus surgery.
61	Intermittent diplopia and aesthenopia	10XP	0	Decompensating XP	20	55	Distance exercises and consideration for surgery
